# Mechanisms and emerging research trends of angiogenesis promotion by small extracellular vesicles from different cellular sources in alleviating myocardial infarction injury

**DOI:** 10.3389/fphar.2026.1766323

**Published:** 2026-03-16

**Authors:** Lian Liu, Hang Hu, Yu Cao, Lu Gan

**Affiliations:** Department of Emergency Medicine, Institute of Disaster Medicine and Institute of Emergency Medicine, West China Hospital, Sichuan University, Chengdu, China

**Keywords:** angiogenesis, endothelial cells, intercellular communication, myocardial infarction, small extracellular vesicles

## Abstract

Myocardial infarction (MI), a lethal coronary artery disease primarily triggered by atherothrombosis or an imbalance in myocardial oxygen supply and demand, stands as a leading cause of mortality worldwide. Promoting angiogenesis is recognized as an effective therapeutic strategy for MI, a process highly dependent on the functional status of endothelial cells (ECs). Small extracellular vesicles (sEVs), which are membrane-bound vesicles secreted by cells and enriched with bioactive molecules including proteins, lipids, and RNAs, are ubiquitously present in the secretome of diverse cell types such as stem cells, immune cells, and cardiac cells. Studies have confirmed that sEVs can deliver specific “cargo” such as miRNAs and cytokines via paracrine or endocrine pathways, activating key downstream signaling cascades. This effectively promotes EC proliferation, migration, and tube formation, thereby enhancing angiogenic capacity and ultimately mitigating pathological cardiac remodeling while improving prognosis post-MI. This review focuses on sEVs derived from various cellular sources, systematically summarizing their roles in promoting angiogenesis and the latest research advances in regulating EC function, aiming to provide novel insights for the effective treatment of MI.

## 1 Introduction

Myocardial infarction (MI) is a severe form of ischemic heart disease. Its primary cause lies in thrombus formation and arterial occlusion, leading to a sudden complete or partial blockage of blood flow to the myocardium, resulting in insufficient cardiac blood supply (Gil-Cabrerizo et al., 2023; Salari et al., 2023). Without timely intervention, MI may progress to malignant arrhythmias, heart failure, or sudden cardiac death, thereby substantially worsening prognosis and quality of life (Feng et al., 2024; Golforoush et al., 2020). In recent years, promoting angiogenesis has become a key therapeutic strategy for ischemic heart disease. After MI, upregulating pro-angiogenic cytokines or receptors in ischemic myocardial tissue can stimulate collateral circulation, restore perfusion to the infarcted area, and improve oxygen and nutrient supply, thereby attenuating the progression of heart failure. This approach is crucial for salvaging jeopardized cardiomyocytes in the peri-infarct zone and improving cardiac function (Berkeley et al., 2023; Martín-Bórnez et al., 2023).

Endothelial cells (ECs), the principal components of the cardiac microcirculation, play a fundamental role in the process of angiogenesis (Wang and Tang, 2021; Anstine et al., 2016). They contribute to new vessel formation through proliferation, migration, and tube formation (Cui et al., 2023; Fu et al., 2023; Gete et al., 2021; Dudley and Griffioen, 2023). Angiogenesis initiation relies on EC activation and migration. The binding of vascular endothelial growth factor (VEGF) to its endothelial receptors activates downstream signaling cascades, including the phosphoinositide 3-kinase/protein kinase B (PI3K/Akt) pathway, which induces pseudopodia formation and basement membrane degradation, thereby facilitating EC migration toward ischemic regions (Rashidi et al., 2024). Subsequently, VEGF and fibroblast growth factor (FGF) further activate mitogenic signaling pathways, promoting EC proliferation. The proliferated ECs adhere via junctional molecules such as VE-cadherin and gradually assemble into tubular structures, forming new vascular lumens (Li et al., 2025). Additionally, ECs facilitate migration by secreting plasminogen activators and matrix metalloproteinases (MMPs) to degrade the extracellular matrix, and release vasoactive substances like nitric oxide (NO) to regulate vascular tone and local blood flow, thereby promoting the stabilization and functionality of new vessels (Li et al., 2025; Krüger-Genge et al., 2019). Hence, modulating EC function is essential for enhancing angiogenesis and improving cardiac repair post-MI.

Following MI, the endogenous pro-angiogenic response is often insufficient to achieve effective revascularization of the ischemic area (Tariq et al., 2022). Therefore, exogenous strategies to promote angiogenesis have become a focus of therapeutic research. Recently, small extracellular vesicles (sEVs) have emerged as promising therapeutic tools in MI treatment. Studies have demonstrated that sEVs regulate multiple aspects of angiogenesis, including the promotion of EC migration, proliferation, and tube formation, as well as the inhibition of apoptosis (Ateeq et al., 2024; Zhu et al., 2023; Pedersen et al., 2024). They carry bioactive molecules such as microRNAs (miRNAs) and proteins derived from donor cells, which can be delivered to ECs via paracrine, endocrine, or autocrine mechanisms, precisely modulating signaling pathways related to angiogenesis. This enhances EC survival, migration, and tube formation under ischemic and hypoxic conditions, ultimately promoting vascular regeneration and perfusion recovery in the infarcted myocardium (Xu et al., 2022; Liu et al., 2023; Matsubara et al., 2021).

It is noteworthy that sEVs derived from different cell types carry distinct sets of signaling molecules and exhibit varied expression profiles, leading to diverse mechanisms in promoting angiogenesis. On this basis, the present review systematically summarizes recent advances in understanding the roles and mechanisms of cell-derived sEVs in post-MI angiogenesis, with the aim of elucidating their therapeutic potential and providing a theoretical foundation for clinical translation.

## 2 Small extracellular vesicles

### 2.1 Biogenesis and biological characteristics of sEVs

**FIGURE 1:**
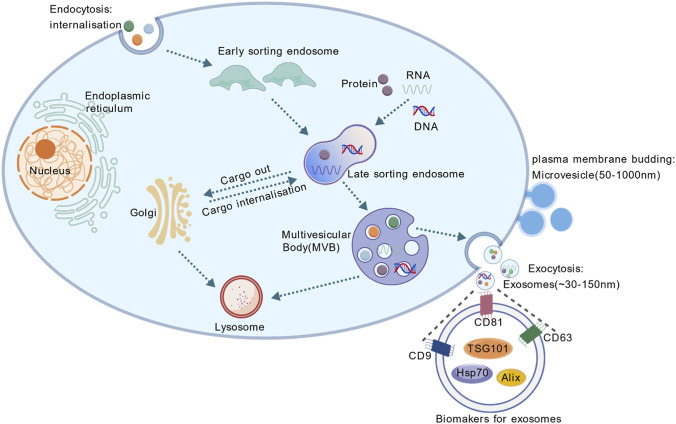
The biogenesis and protein markers of small extracellular vesicles (sEVs). Initially, the plasma membrane undergoes inward invagination to encapsulate extracellular components and membrane proteins, forming early endosomes. Subsequently, the endosomal membrane of these early endosomes experiences multiple invaginations. During this maturation phase, early endosomes not only engage in material exchange with other organelles but may also undergo fusion events, ultimately developing into late endosomes. The final stage involves the fusion of late endosomes with the plasma membrane, resulting in the extracellular release of intraluminal vesicles through exocytosis - these secreted vesicles are recognized as exosomes. Microvesicles are released directly from the plasma membrane to the outside of the cell. Extracellular vesicles smaller than 300 nm are collectively referred to as sEVs.

Extracellular vesicles (EVs) are crucial signal transduction mediators within the body, facilitating communication between different cells and organs. Exosomes (Exo, 30–150 nm) and microvesicles (MVs, 50–1,000 nm), which arise from distinct biogenetic pathways, constitute the two principal subtypes of EVs (Mathieu et al., 2019; Sluijter et al., 2018) (Figure 1). Owing to the limitations of current isolation techniques, EVs smaller than 300 nm obtained through conventional approaches are collectively referred to as sEVs (Sluijter et al., 2018). In recent years, sEVs have been recognized as novel signaling vehicles that mediate intercellular and inter-organ communication through their cargo (Thomou et al., 2017; Kalluri and LeBleu, 2020).

sEVs are membrane-bound vesicles characterized by a highly stable lipid bilayer structure (Yang et al., 2020). Under transmission electron microscopy, sEVs typically exhibit a classic cup-shaped or saucer-like morphology. They are further characterized by specific surface protein markers, including tumor susceptibility gene 101 (TSG101), Alix, heat shock protein 70 (HSP70), and the tetraspanins CD63 and CD81, among others (Feng et al., 2024; Aguilar et al., 2023). These sEVs are abundantly present in various mammalian bodily fluids such as urine, plasma, breast milk, and cerebrospinal fluid, and are secreted by a wide range of cell types, including cardiomyocytes, ECs, macrophages, dendritic cells, and stem cells (Nian and Fu, 2023; Tschuschke et al., 2020). sEVs carry diverse bioactive cargo—such as proteins, lipids, DNA, mRNAs, miRNAs, and long non-coding RNAs (lncRNAs)—that reflect the pathophysiological state of their parent cells. They play a crucial role in intercellular communication via autocrine, paracrine, and endocrine mechanisms. By transferring their contents to recipient cells, sEVs facilitate signal transduction and modulate cellular functions, thereby contributing significantly to various physiological and pathological processes (Gai et al., 2016; Busatto et al., 2021; Kanada et al., 2015; Hao et al., 2020).

### 2.2 sEVs and MI

sEVs, as key mediators of intercellular communication, exhibit substantial therapeutic potential in diverse pathological conditions, including cancer, renal disorders, inflammatory diseases, and neurodegenerative disorders (Zhang et al., 2022a; Thongboonkerd, 2020; Yu et al., 2021; Ma et al., 2023; Pulliam et al., 2019). Research on sEVs in the cardiovascular field is advancing rapidly, particularly regarding their involvement in multiple stages following MI. sEVs are involved in both the initiation and progression of MI. They modulate multiple pathophysiological processes, including cardiomyocyte necrosis and apoptosis, angiogenesis, suppression of inflammatory responses, and myocardial fibrosis (Henning, 2021; Yang, 2018; Fu et al., 2020). In addition to the regulation mediated by sEVs, endogenous cytokines within the myocardial injury microenvironment also serve as critical drivers for activating key repair pathways. For example, interleukin-33 (IL-33) released by cardiomyocytes has been demonstrated to effectively induce angiogenesis by directly activating the AKT/eNOS signaling pathway in endothelial cells (Yu et al., 2024). sEVs derived from various cell sources represent a potential strategy for MI repair, with their core mechanisms closely associated with angiogenesis and the regulation of signaling pathways. Existing research indicates that targeted activation of the PI3K/AKT/mTOR pathway can effectively ameliorate myocardial dysfunction, as demonstrated by Sivelestat in the treatment of sepsis-induced myocardial injury (Geng et al., 2024). sEVs can also promote angiogenesis and provide myocardial protection following MI by modulating this pathway. Serum-sEVs from ischemic preconditioned rats activate the PI3K/Akt pathway, upregulate B-cell cll/lymphoma 2 (BCL-2), phosphorylated phosphoinositide 3-kinase (p-PI3K), and phosphorylated protein kinase B (p-AKT), and suppress caspase-3 and Bax expression, resulting in decreased inflammatory cytokine release and cardiomyocyte apoptosis, ultimately improving cardiac function (Zhang and Zhang, 2021). Cardiac telocytes are a novel type of interstitial cells recently identified within the cardiac stroma. Liao et al. demonstrated that sEVs derived from cardiac telocytes carry miRNA-21-5p, which inhibits apoptosis in microvascular ECs by targeting the cell death-inducing p53 target 1(CDIP1) gene and reducing activated caspase-3 levels, thereby promoting vascular regeneration after infarction (Liao et al., 2021). Similarly, Zhu et al. reported that sEVs from umbilical cord mesenchymal stem cells(UC-MSCs-sEVs), enriched with macrophage migration inhibitory factor, markedly enhance the proliferation, migration, and tube formation of human umbilical vein ECs (HUVECs) under ischemic and hypoxic conditions. These sEVs also inhibit apoptosis in H9C2 cardiomyocytes, with miRNA-133a-3p identified as a critical mediator that mitigates apoptosis and myocardial fibrosis while enhancing cardiac function (Zhu et al., 2021). Additional studies have revealed that adipose-derived stem cell sEVs (ADSC-sEVs) containing miR-221/222 mitigate myocardial injury in MI models by downregulating apoptotic markers (p-p53, PUMA) and hypertrophic markers (ETS-1, ANP) (Lai et al., 2020). sEVs from M2 macrophages deliver miR-148a, which suppresses interleukin-1 β (IL-1β) and interleukin-18 (IL-18) through inhibition of thioredoxin interacting protein (TXNIP) and the toll-like receptor 4)/nuclear factor κ-light-chain-enhancer of activated B Cells/nod-like receptor pyrin domain containing 3 ((TLR4/NF-κB/NLRP3) inflammasome pathway, leading to reduced infarct size, improved myocardial enzyme profiles, and attenuated Ca^2+^ overload in a rat MI model (Dai et al., 2020).

The therapeutic efficacy of sEVs-based interventions for MI has been substantiated by extensive experimental evidence. sEVs have been successfully isolated from diverse sources—such as rat plasma, rat bone marrow mesenchymal stem cells (BM-MSCs), embryonic stem cells (ESCs), and hUC-MSCs—and applied in MI treatment models (Jin et al., 2021; Sun et al., 2020; Kesidou et al., 2024; Shao et al., 2023). Studies consistently indicate that sEVs from these varied origins produce comparable and substantial therapeutic benefits, including improved cardiac function, reduced infarct size, enhanced angiogenesis, and inhibition of cardiomyocyte apoptosis. Collectively, these findings expand the spectrum of potential sEV sources and isolation strategies, while reinforcing the foundation for their clinical translation and offering promising avenues for MI therapy.

## 3 Effects of sEVs from different cellular sources on angiogenesis after MI

### 3.1 Stem cell-derived sEVs

#### 3.1.1 sEVs derived from mesenchymal stem cells

Mesenchymal stem cells (MSCs), characterized by self-renewal and multilineage differentiation potential, are distributed across various tissues and body fluids, including bone marrow, umbilical cord blood, adipose tissue, and urine (Ren et al., 2020; Nazari-Shafti et al., 2020). Substantial evidence underscores the pivotal role of MSC-sEVs in enhancing angiogenesis and promoting functional recovery post-MI. For example, rBM-MSC-sEVs upregulate hypoxia-inducible factor 1 α (HIF-1α), thereby increasing the expression of pro-angiogenic factors such as VEGF and platelet-derived growth factor (PDGF) and promoting the migration, proliferation, and tube formation of hypoxia-injured HUVECs (Sun et al., 2020). Similarly, hBM-MSC-sEVs carrying miR-543 enhance the proliferation, migration, invasion, and tube formation of cardiac microvascular ECs *via* downregulation of collagen type IV alpha 1 chain (COL4A1), mitigating MI-induced injury (Yang et al., 2021). Upregulation of miR-29b-3p in MSC-sEVs has been shown to improve myocardial angiogenesis and ameliorate ventricular remodeling in a rat MI model by targeting a disintegrin and metalloproteinase with thrombospondin type 1 motif 16 (ADAMTS16), resulting in reduced fibrosis, decreased collagen volume fraction, elevated capillary density, and increased VEGF expression, collectively supporting endothelial proliferation and tube formation (Zheng et al., 2022).

HUC-MSC-sEVs deliver miR-214, which promotes EC proliferation by inhibiting suppressor of fused homolog (Sufu) and activating the hedgehog pathway in HUVECs, thereby stimulating neovascularization and facilitating cardiac repair (Shao et al., 2023). Moreover, miR-423-5p encapsulated in hUC-MSC-sEVs enhances EC migration and tube formation by targeting Ephrin A3 (EFNA3) (Gao et al., 2022). Pretreating hUC-MSCs with nicotinamide mononucleotide (NMN) enriches miR-210-3p in their sEVs, markedly enhancing EC angiogenic activity through ephrin a3 suppression and promoting myocardial recovery (Pu et al., 2023). sEVs from interferon-γ-stimulated (IFN-γ) hUC-MSCs elevate miR-21, enhancing the migration and tube formation of oxygen-glucose deprived (OGD) HUVECs and attenuating apoptosis in H9C2 cells by suppressing B-cell translocation gene 2 (BTG2) (Zhang J. et al., 2022). Under oxygen-glucose deprived conditions, Huc-MSC-sEVs elevate miR-1246, which inhibits serine protease 23 (PRSS23) and snail family zinc finger 1/α-smooth muscle actin (Snail/α-SMA) signaling, upregulating VEGFA and CD31 to promote EC proliferation, tube formation, and functional vascularization (Wang et al., 2021). Notably, the reparative potential of hucMSCs extends beyond exosomes. Their derived apoptotic bodies have also demonstrated efficacy in a large animal model of myocardial infarction. These particles encapsulate more complex cellular contents, which, upon uptake by phagocytic cells in the infarcted area, potently modulate immune responses and promote repair, thereby improving cardiac function and structure (Luo et al., 2025). This reveals that exosomes and apoptotic bodies together constitute a complementary acellular therapeutic system. Furthermore, sEVs from mouse cardiac tissue-derived MSCs enhance EC tube formation via Notch1 overexpression, facilitating dense tubular network formation *in vitro* and augmenting angiogenesis in peri-infarct regions *in vivo* (Xuan et al., 2020). sEVs derived from dental pulp mesenchymal stem cells carrying miR-4732-3p significantly promote tube formation in HUVECs and enhance vascularization in nude mice, underscoring the role of MSC-sEVs in tube formation and angiogenic repair following ischemic injury (Sánchez-Sánchez et al., 2021).

Beyond directly regulating endothelial cell function, MSC-sEVs can also promote post-infarction angiogenesis indirectly by remodeling the local microenvironment through targeting multiple cell types. On one hand, MSC-sEVs engineered to carry active mitochondria can be internalized by macrophages. By improving mitochondrial function in these cells, they drive the polarization from the pro-inflammatory M1 phenotype towards the reparative M2 phenotype. The M2 macrophages subsequently secrete factors that foster an immune microenvironment conducive to angiogenesis (Dong et al., 2025). On the other hand, sEVs secreted by MSCs pretreated with the drug Vericiguat are enriched with miR-1180-3p. Upon uptake by cardiac fibroblasts, these sEVs inhibit the ever shorter telomeres 1 (ETS1)signaling pathway, thereby suppressing fibroblast activation and excessive collagen deposition. This alleviates pathological fibrosis and provides a permissive physical matrix for nascent vessel extension and maturation (Li C. et al., 2024). In conclusion, by coordinately modulating macrophages and fibroblasts, MSC-sEVs optimize the angiogenic microenvironment from both immunological and structural perspectives, representing a pivotal indirect mechanism for promoting cardiac repair.

**FIGURE 2:**
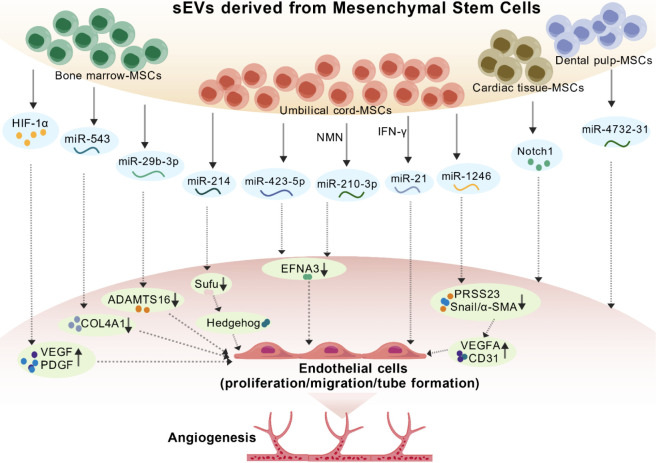
Mechanisms involved in the modulation of angiogenesis by MSC-derived sEVs. BM-MSC-sEVs deliver HIF-1α, miR-543, and miR-29b-3p, which promote EC proliferation, migration, and tube formation *via* downstream signaling pathways involving VEGF, COL4A1, and ADAMTS16, respectively. UC-MSC-sEVs carry miR-214 and miR-423-5p, exerting their functions by targeting Sufu and EFNA3, respectively. Additionally, UC-MSC-sEVs pretreated with NMN or IFN-γ can also deliver pro-angiogenic miRNAs. sEVs from mouse cardiac tissue-derived MSCs secrete miR-1246, Notch1 which acts on ECs. Dental pulp mesenchymal stem cell-derived sEVs deliver miR-4732-3p, directly promoting EC proliferation, migration, and tube formation, ultimately facilitating angiogenesis. VEGF, vascular endothelial growth factor; PDGF, platelet-derived growth factor; COL4A1, collagen type IV alpha 1 chain; ADAMTS16, a disintegrin and metalloproteinase with thrombospondin type 1 motif 16; Sufu, fused homolog; EFNA3 Ephrin A3; PRSS23, serine protease 23; Snail/α-SMA, snail family zinc finger 1/α-smooth muscle actin; NMN, nicotinamide mononucleotide; IFN-γ, interferon-γ; HIF-1α, hypoxia-inducible factor 1α.

Collectively, the delivery of a spectrum of key miRNAs by MSC-sEVs mediates the regulation of multiple pro-angiogenic signaling pathways, such as HIF-1α/VEGF, hedgehog, and ephrin, which collectively constitute the molecular basis of their robust pro-angiogenic activity (Figure 2). While the aforementioned studies have convincingly demonstrated the therapeutic potential of MSC-sEVs in post-MI repair, the literature indicates that their functional properties are not only significantly influenced by tissue origin but, more critically, also dependent on specific preconditioning protocols employed to enhance their efficacy (e.g., hypoxic, IFN-γ, NMN). These preconditioning approaches enhance sEVs activity through global modulation of their proteomic and miRNA profiles. However, different preconditioning strategies vary in their mechanisms, risks, and impacts on the native characteristics of sEVs. NMN preconditioning represents a metabolic engineering strategy that mildly optimizes sEVs cargo with favorable safety, though its protocol requires further refinement (Pu et al., 2023). IFN-γ preconditioning enhances immunomodulatory and anti-apoptotic functions, but requires careful control to avoid potential pro-inflammatory skewing (Zhang J. et al., 2022). Hypoxia preconditioning directly mimics ischemia and strongly activates pro-angiogenic programs, yet may concurrently induce cellular stress responses (Wang et al., 2021). All these treatments can alter sEVs yield, membrane protein composition, and cargo profiles. Moreover, their reproducibility is constrained by multiple variables, including cell source, preconditioning parameters (e.g., concentration, duration), and isolation methods. The lack of standardized operating procedures currently represents a major bottleneck for achieving reproducible production and reliable clinical translation of MSC-sEVs. Therefore, future research should focus on establishing preconditioning standards and applying multi-omics approaches to comprehensively evaluate sEVs property changes, thereby advancing their translation into clinical products.

#### 3.1.2 sEVs derived from induced pluripotent stem cells

**FIGURE 3:**
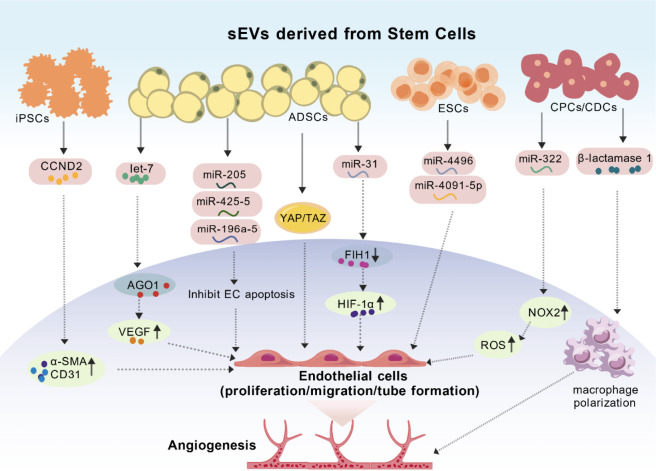
Mechanisms involved in the modulation of angiogenesis by Stem Cell-derived sEVs. IPSC-sEVs secrete CCND2, which promotes the expression of α-SMA and CD31, thereby enhancing EC proliferation, migration, and tube formation. ADSC-sEVs carry multiple signaling molecules (including let-7, miR-205, miR-425-5p, miR-196a-5p, and miR-31) to exert their effects, and can also enhance the glycolytic capacity of ECs *via* activation of the YAP/TAZ pathway. ESC-sEVs promote angiogenesis through the transfer of miR-4496 and miR-4091-5p. CPC-sEVs, when modified with miR-322, act on ECs by increasing NOX2-derived ROS species; CDC-sEVs can also promote macrophage polarization toward a pro-angiogenic phenotype *via* β-lactamase 1. IPSC-sEVs, sEVs from induced pluripotent stem cells; ESCs-sEVs, sEVs from embryonic stem cells; CPCs/CDCs, sEVs from cardiac stem/progenitor cells; CCND2, cyclin D2; ADSC-sEVs, sEVs from adipose-derived stem cell; let-7/AGO1/VEGF, let-7 microRNA/Argonatute 1/vascular endothelial growth factor axis; YAP/TAZ, yes-associated protein/transcriptional co-activator with pdz-binding motif; ESC-sEVs, sEVs Derived from Embryonic Stem Cells; ROS, reactive oxygen species; FIH1, inhibiting factor inhibiting HIF 1; NOX_2_, NADPH oxidase 2.

Induced pluripotent stem cells (iPSCs), generated through somatic cell reprogramming, exhibit robust self-renewal and pluripotency. iPSC-derived sEVs (iPSC-sEVs) exhibit pronounced pro-angiogenic effects, underscoring their therapeutic potential in MI (Figure 3). In a porcine MI model, sEVs from iPSC-derived cardiomyocytes overexpress cyclin D2 (CCND2), upregulating α-SMA and CD31 and substantially promoting angiogenesis (Zhao et al., 2021). Gao et al. confirmed that iPSC-sEVs enhance EC tube formation and myocardial recovery in both *in vivo* and *in vitro* MI models (Gao et al., 2020a). Additionally, sEVs from iPSC-derived cardiomyocytes are enriched with angiogenesis-related miRNAs and proteins that stimulate EC proliferation, neovascularization, and anti-fibrotic responses, underscoring their potential for treating ischemic heart disease (Tominaga et al., 2024). Moreover, sEVs released from induced pluripotent stem cells and enriched with miR-126 can enhance the expression of angiogenic factors, thereby facilitating endothelial cell-associated angiogenesis (Kmiotek et al., 2024). Although previous studies have elucidated the pro-angiogenic functions of iPSC-sEVs in MI, the regulatory mechanisms by which iPSC-sEVs precisely package angiogenesis-related molecules (e.g., CCND2 and specific miRNAs) during the differentiation of iPSCs into cardiomyocytes remain to be fully elucidated—clarifying this process would facilitate the optimization of strategies for “engineering” iPSC-sEVs with enhanced pro-angiogenic potency.

#### 3.1.3 sEVs derived from embryonic stem cells

ESCs and iPSCs share fundamental biological properties, including self-renewal and multilineage differentiation capacity. In MI research, ESC-sEVs have demonstrated beneficial effects (Figure 3). sEVs from human embryonic stem cell-cardiovascular progenitor cells (hESC-CVPC-sEVs) carrying the long non-coding RNA metastasis associated lung adenocarcinoma transcript 1 (MALAT1) promote infarct repair through the MALAT1/miR-497 axis, which coordinately protects cardiomyocytes, inhibits fibroblast activation, and drives macrophage polarization toward the M2 phenotype. This integrated action reduces apoptosis and fibrosis, enhances the anti-inflammatory response, and stimulates the secretion of endogenous pro-angiogenic factors, thereby multi-directionally remodeling the infarct microenvironment to facilitate vascular network reconstruction (Wu et al., 2020). Studies reveal that sEVs secreted from hESC-derived ECs are enriched with miR-4496 and miR-4691-5p. Under *in vitro* conditions, these sEVs significantly promote EC tube formation and wound healing, highlighting their potent pro-angiogenic function (Kesidou et al., 2024). Furthermore, comparative studies on sEVs from different cellular sources for post-MI cardiac repair indicate that those secreted by hESCs exhibit notably enhanced pro-proliferative and pro-tubulogenic effects on ECs *in vitro* (González-King et al., 2024). However, It remains unclear whether the pro-angiogenic effects of ESC-sEVs—particularly those mediated by miR-4496 and miR-4691-5p—can be sustained in long-term *in vivo* MI models, rather than merely observed in short-term *in vitro* assays, as well as what their clinical translation potential entails.

#### 3.1.4 sEVs derived from adipose-derived stem cells

ADSCs, a mesenchymal stem cell subtype, support angiogenesis through direct differentiation into EC and vascular smooth muscle cells (VSMCs), as well as *via* paracrine sEVs-mediated mechanisms (Yang et al., 2024; Deveza et al., 2013) (Figure 3). Studies demonstrate that ADSC-sEVs enhance EC proliferation, migration, tube formation, and VEGF secretion under both normoxic and hypoxic conditions, primarily *via* activation of the let-7 microRNA/Argonaute 1/Vascular Endothelial Growth Factor axis (let-7/AGO1/VEGF) (Zhu et al., 2020; Han C. et al., 2019). MicroRNAs within ADSC-sEVs, particularly let-7 family members, significantly improve EC migration and invasion (Huang et al., 2019). Within ADSC-sEVs, specific microRNAs such as miR-205, miR-196a-5p, miR-425-5p, and miR-31 have been identified as key initiators of cardiac angiogenic pathways, forming a crucial basis for ADSC-sEVs-mediated mitigation of MI injury and promotion of cardiac functional recovery. Specifically, MiR-205 promotes microvascular EC proliferation and migration while inhibiting cardiomyocyte apoptosis (Wang et al., 2023). miR-196a-5p and miR-425-5p attenuate mitochondrial dysfunction and oxidative stress, suppress EC apoptosis, and enhance tube formation (de Almeida Oliveira et al., 2022). miR-31 facilitates HIF-1α nuclear translocation and transcriptional activity by inhibiting factor inhibiting HIF 1 (FIH1), promoting EC migration and tube formation (Zhu D. et al., 2022). Glycolysis, the principal metabolic pathway supplying energy to vascular endothelial cells, plays a pivotal role in angiogenesis. Studies have shown that ADSC-sEVs enhance glycolytic flux and suppress oxidative phosphorylation in ECs *via* yes-associated protein/transcriptional co-activator with Yes-associated protein/Transcriptional coactivator with PDZ-binding motif (YAP/TAZ) activation, stimulating angiogenic processes (Sun J. et al., 2023). ADSC-sEVs not only directly promote angiogenesis by regulating EC metabolism but also indirectly optimize the angiogenic microenvironment by remodeling the extracellular matrix. Specifically, active components carried by ADSC-sEVs, such as antioxidant proteins, exert a dual regulatory effect by suppressing the expression of activating transcription factor 3 (ATF3) in cardiac fibroblasts. This inhibition reduces collagen synthesis and deposition, thereby attenuating pathological fibrosis, while simultaneously promoting the secretion of pro-angiogenic factors—including hepatocyte growth factor (HGF) and VEGF—from fibroblasts. The concurrent remodeling of the matrix and supply of growth factors collectively establish essential conditions for the formation of a functional vascular network (Chen et al., 2025). Consequently, this effectively promotes the proliferation, migration, invasion, and tube formation abilities of ECs. This research provides novel insights and therapeutic strategies for the field of tissue regeneration related to revascularization. In light of these multifaceted mechanisms, ADSC-sEVs emerge as a promising cell-free regenerative strategy, warranting further investigation into their therapeutic development for ischemic diseases.

#### 3.1.5 sEVs derived from cardiac stem/Progenitor cells

Cardiac progenitor cells (CPCs), occupying an intermediate niche between stem and somatic cells, differentiate into multiple cardiac lineages. Currently, existing studies have shown that CPCs exert potent cardioprotective effects in MI through multiple molecular pathways (Figure 3). CPC-sEVs promote the proliferation and differentiation of regulatory T cells (Tregs) by activating the mechanistic target of rapamycin (mTOR) signaling pathway. The resultant expansion of the Treg population systemically suppresses excessive inflammatory responses, thereby creating an immune-tolerant microenvironment conducive to reparative processes such as angiogenesis (He et al., 2023). CPC-sEVs enhance EC migration and tube formation, particularly under moderate hypoxia (5% O_2_), highlighting the therapeutic utility of hypoxia-preconditioned human CPC-sEVs (Dougherty et al., 2020). Bioengineered CPC-sEVs loaded with miR-322 augment EC migration and capillary formation *via* NADPH oxidase 2(NOX_2_)-derived reactive oxygen species (ROS), offering a promising strategy for ischemic cardiovascular therapy (Youn et al., 2019). Studies in large animals have indicated that in porcine models of MI, when clinical-grade CPC-sEVs are administered via intracoronary injection within 15 min after reperfusion, molecules such as miR-132 contained in these sEVs can activate EC migration and tube formation, thereby promoting neovascularization in the infarcted area (Emmert et al., 2024). Cardiosphere-derived cells (CDCs), another progenitor population, generate ECs, cardiomyocytes, and smooth muscle cells. CDC-sEVs promote macrophage polarization toward a pro-angiogenic phenotype via β-lactamase 1 upregulation, enhancing angiogenesis and immunomodulation (Mentkowski et al., 2020). Collectively, these findings position CPC-sEVs as promising next-generation therapeutics, with their efficacy potentially optimized through bioengineering strategies and preconditioning protocols.

#### 3.1.6 sEVs derived from various stem cells: differences and similarities

sEVs from various stem cell sources share a core mechanism: microenvironment-responsive delivery of bioactive molecules. Through miRNAs (e.g., miR-214, miR-205) and proteins (e.g., HIF-1α, CCND2), they modulate pathways such as VEGF, HIF-1α, and PI3K/Akt, enhancing EC proliferation, migration, and angiogenic function, ultimately improving perfusion in infarcted areas(Figures 2, 3; Table 1).

**TABLE 1:** Summary of studies on stem cell-derived sEVs promoting angiogenesis after MI.

sEVs Cell source	Key molecule(s)	Mechanism of action	Model validation	Relative advantages and disadvantages	References
Mesenchymal stem cells (bone Marrow)	HIF-1α	Activates VEGF/PDGF pathways, promotes endothelial cell (EC) proliferation/migration/tube formation.	*In vitro*: Human umbilical vein endothelial cells (HUVECs). *In vivo*: Rat MI model.	Advantages: 1. Wide sources (bone marrow, umbilical cord, adipose, *etc.*); 2. Strong immunomodulatory capacity, potential for allogeneic application; 3. Relatively high safety, no reported tumorigenicity. Disadvantages: 1. High heterogeneity, functions affected by tissue source, donor, and culture conditions; 2. Difficulties in standardization for large-scale production.	Sun et al. (2020)
	miR-543	Downregulates COL4A1, promotes EC proliferation/migration/tube formation.	*In vitro*: Cardiac microvascular endothelial cells (CMECs). *In vivo*: Rat MI model.		Yang et al. (2021)
	miR-29b-3p	Targets ADAMTS16 to inhibit fibrosis and upregulate VEGF expression.	*In vitro*: Endothelial cells (HUVECs/RCMECs). *In vivo*: Rat MI model.		Zheng et al. (2022)
Mesenchymal stem cells (umbilical cord)	miR-214	Activates Sufu/hedgehog pathway, promotes EC proliferation/migration/tube formation.	*In vitro*: HUVECs. *In vivo*: Rat MI model.		Shao et al. (2023)
	miR-423-5p	Targets ephrin A3 (EFNA3) to enhance endothelial cell migration and lumen formation.	*In vitro*: HUVECs. *In vivo*: Rat MI model.		Gao et al. (2022)
	miR-210-3p	Inhibits EFNA3, enhances angiogenic activity of endothelial cells.	*In vitro*: HUVECs. *In vivo*: Rat MI model.		Pu et al. (2023)
	miR-1246	Inhibits PRSS23/Snail/α-SMA pathway, upregulates VEGFA and CD31.	*In vitro*: HUVECs. *In vivo*: Rat heart failure model.		Wang et al. (2021)
Induced pluripotent stem cells	CCND2	Upregulates α-SMA and CD31 expression, promotes angiogenesis.	*In vitro*: Co-culture of hiPSC-derived cardiomyocytes and endothelial cells. *In vivo*: Porcine MI model.	Advantages: 1. No ethical restrictions, with potential for autologous sourcing; 2. Potent functionality, as vesicle cargo may retain partial pluripotency-associated characteristics. Disadvantages: 1. Potential tumorigenic risk (lower than that of the parent cells but requiring long-term validation); 2. Long autologous preparation cycle, which is less suitable for acute therapeutic applications.	Zhao et al. (2021)
Embryonic stem cells	miR-4496, miR-4691-5p	Directly promotes endothelial cell wound healing and lumen formation.	*In vitro*: HUVECs. *In vivo*: Mouse matrigel plug or hindlimb ischemia model.	Advantages: Potentially the strongest pro-angiogenic ability due to the most primitive origin. Disadvantages: 1. Ethical controversies; 2. Immune rejection issues.	Kesidou et al. (2024)
Adipose-derived stem cells	Let-7 family members	Activates argonaute 1/VEGF pathway, promotes EC proliferation/migration/tube formation.	*In vitro*: HUVECs. *In vivo*: Immunodeficient mouse subcutaneous co-transplantation model of sEVs with fat granules.	Advantages: 1. Extremely abundant source, minimal harvesting trauma; 2. Potential for direct differentiation into vascular cells. Disadvantages: 1. Adipose tissue composition is complex, sEVs extraction requires strict removal of non-target cell contamination; 2. Donor metabolic health status (e.g., obesity, diabetes) may affect sEVs quality.	Zhu et al. (2020)
	miR-31	Inhibits FIH1, promotes HIF-1α nuclear translocation and transcriptional activity, enhances EC migration and lumen formation.	*In vitro*: HUVECs. *In vivo*: Mouse MI model; hindlimb ischemia (HLI) model.		Zhu D. et al. (2022)
Cardiac stem/Progenitor cells	miR-322	Increases NOX2/ROS, promotes endothelial cell migration.	*In vitro*: Vascular endothelial cells. *In vivo*: Mouse myocardial ischemia/reperfusion model.	Advantages: 1. High tissue specificity, homing and repair signals may be more precise; 2. Possesses novel immunomodulatory mechanisms (e.g., macrophage reprogramming) Disadvantages: 1. Limited source, difficult to obtain from cardiac tissue; 2. Relatively scarce preclinical *in vivo* data, insufficient validation.	Youn et al. (2019)
	β-lactamase 1	Modulates macrophage polarization towards a pro-angiogenic phenotype.	*In vitro*: HUVECs. *In vivo*: Mouse MI model.		Mentkowski et al. (2020)

Different sEVs sources each possess distinct advantages: MSC-sEVs are widely available and mechanism-specialized; ADSC-sEVs combine direct differentiation and paracrine signaling; CPC-sEVs show microenvironment dependency and immunomodulatory capacity; iPSC-sEVs and ESC-sEVs offer additional anti-fibrotic and anti-inflammatory benefits. However, several limitations remain. First, most studies focus on sEVs from a single stem cell type, leaving unexplored the potential synergies between sEVs from different sources (e.g., whether combined administration of ADSC-sEVs and CPC-sEVs yields additive effects). Second, current research relies predominantly on small-animal models (e.g., mice and rats), with limited validation in large-animal or preclinical settings. This limits translational relevance to the complex pathological progression of human MI.

To address these gaps, future studies should prioritize: (1) optimizing sEVs potency through preconditioning strategies such as hypoxia or NMN; (2) designing combination therapies leveraging complementary sEVs properties from different cellular origins; and (3) conducting long-term follow-up studies in large animal models to strengthen the evidence base for clinical application.

### 3.2 Non-stem cell-derived sEVs

#### 3.2.1 sEVs derived from cardiac cells

##### 3.2.1.1 sEVs derived from cardiomyocytes

Cardiomyocytes package abundant non-coding RNAs into sEVs that are internalized by neighboring ECs, enhancing EC proliferation, migration, and tube formation. This intercellular communication supports angiogenesis and reduces fibrosis after MI (Liu et al., 2023). In numerous research instances, hypoxic cardiomyocytes have been found to release sEVs enriched with circular homeodomain-interacting protein kinase 3 RNA (circHIPK3). This circular RNA mitigates oxidative stress-induced injury in cardiac endothelial cells by sponging miR-29a, which subsequently leads to increased expression of VEGFA. Ultimately, this process promotes the proliferation, migration, and tube formation of cardiac endothelial cells (Wang et al., 2020). Additionally, Chen et al. reported that miR-146a-5p derived from cardiomyocytes not only promotes macrophage polarization, endowing them with a dual regulatory capacity encompassing both pro-inflammatory and anti-inflammatory phenotypes, but also upregulates the expression of VEGFA. This elevated VEGFA level subsequently enhances ROS production and augments autophagy mediated by endoplasmic reticulum stress in the infarcted area, collectively promoting EC proliferation, migration, and tube-forming capacity. These mechanisms robustly augment vascular regeneration following cardiac ischemic injury (Chen et al., 2022). Collectively, these findings underscore the pivotal role of sEVs derived from cardiomyocytes as central coordinators of cardiac repair, orchestrating angiogenesis through the delivery of multiple non-coding RNA species that target complementary pathways.

##### 3.2.1.2 sEVs derived from fibroblasts

Cardiac fibroblasts (CFs), constituting up to one-third of the heart’s cellular volume, are essential for maintaining homeostasis and repair (Henning, 2021). Their sEVs promote angiogenesis and inhibit cardiomyocyte apoptosis *via* stromal cell-derived factor-1 (SDF-1) and VEGF secretion (Tseliou et al., 2015).

Endothelial pas domain protein 1 (Epas1) is a transcription factor that regulates the expression of VEGF. Studies have confirmed that Epas1 is highly expressed in fibroblasts of adult mice, and this high expression enables it to exhibit a significant proangiogenic effect. Given that a core characteristic of sEVs is their ability to carry bioactive signaling molecules from their parent cells, it can be inferred that fibroblast-sEVs may also contain highly expressed Epas1 and thus potentially possess significant proangiogenic capacity (Sun H. et al., 2023). This compelling hypothesis, however, awaits direct validation through further experiments, such as those utilizing Epas1 knockdown or overexpression models. Studies using dermal fibroblasts cultured on 3D silk fibroin scaffolds show that sEVs containing angiopoietins (Ang-1/Ang-2), Interleukin 1-1α (IL-1α), and interleukin 8 (IL-8) enhance EC migration and tube formation *via* PI3K/Akt and MAPK/ERK pathways (Hu et al., 2023). Conversely, CFs expressing high levels of ly6/plaur domain-containing protein 1 (LYPD-1) inhibit angiogenesis. LYPD-1-rich sEVs may suppress EC function, indicating that LYPD-1 inhibition could represent a therapeutic strategy for enhancing angiogenesis (Sakamoto et al., 2020). In summary, CF-sEVs play a complex, dual regulatory role in cardiac angiogenesis, influenced by their specific cargo. Notably, three-dimensional culture systems can enhance their pro-angiogenic potential, providing a research direction for improving cardiac angiogenesis by targeting the balance of factors within fibroblast-sEVs.

##### 3.2.1.3 sEVs derived from smooth muscle cell

Smooth muscle cells (SMCs)-based therapies have shown clear potential in the field of tissue regeneration, particularly for ischemic diseases. These cells play a pivotal role in maintaining vascular stability by regulating vascular structural integrity and functional homeostasis (Joo et al., 2014). Research has revealed that sEVs secreted by adult VSMCs cultured on three-dimensional silk fibroin nonwoven scaffolds are enriched with pro-angiogenic factors (e.g., Angiopoietin) and growth-promoting factors. These sEVs effectively promote the proliferation, migration, and tube formation of vascular endothelial cells, offering a promising sEVs source and application strategy for vascular injury repair and the treatment of ischemic diseases (Hu P. et al., 2022). Furthermore, sEVs derived from iPSCs-differentiated SMCs can also promote EC migration, proliferation, and differentiation via paracrine mechanisms, exhibiting therapeutic efficacy in therapeutic angiogenesis (Park et al., 2021). Collectively, these findings underscore the multifaceted applications of SMCs and their sEVs in regenerative medicine, providing both a mechanistic foundation and practical strategies for the treatment of ischemic conditions.

#### 3.2.2 sEVs derived from immune cells

##### 3.2.2.1 sEVs derived from macrophages

Macrophages, as essential components of the innate immune system, can undergo phenotypic polarization in response to dynamic changes in the inflammatory microenvironment, primarily differentiating into pro-inflammatory M1 or anti-inflammatory M2 phenotypes (Zhao et al., 2019). Following MI, M2 macrophages contribute to the suppression of inflammatory responses and facilitate a microenvironment conducive to angiogenesis (Peet et al., 2020). sEVs secreted by M2 macrophages (M2-sEVs) play a central role in this process. Enriched with various growth factors and anti-inflammatory cytokines—such as VEGF and PDGF—these sEVs act directly on ECs to promote proliferation, migration, and tube formation, thereby enhancing angiogenesis (Zhu F. et al., 2022). Furthermore, M2-sEVs deliver miR-132-3p into ECs. By downregulating thrombospondin-1 (THBS1), this miRNA enhances endothelial proliferation, migration, and tube formation, thereby strongly promoting post-infarction angiogenesis (Guo et al., 2024). Another study demonstrated that sEVs derived from macrophages transfected with growth differentiation factor 15 (GDF-15) activate mothers against decapentaplegic homolog 2/3 (Smad2/3) phosphorylation and subsequently inhibit fatty acid-binding protein 4 (FABP4) production. This mechanism enables precise modulation of macrophage phenotype, inhibits cardiomyocyte apoptosis, and significantly enhances EC proliferation, migration, and tube-forming capacity (Xiao et al., 2024). In addition, M2 macrophage-derived sEVs deliver miR-148a to promote angiogenesis by dual immunomodulation: inhibiting the TXNIP/TLR4/NF-κB/NLRP3 inflammasome in neutrophils to curb inflammation, and polarizing macrophages to the M2 phenotype to boost VEGF/HGF secretion, thereby fostering a pro-reparative milieu (Dai et al., 2020). Therefore, M2-sEVs represent a promising cell-free therapeutic platform that leverages inherent immunomodulatory and pro-angiogenic cargo to orchestrate multi-faceted repair after MI.

##### 3.2.2.2 sEVs derived from dendritic cells

Dendritic cells (DCs), as pivotal antigen-presenting cells, play an essential immunomodulatory role following MI (Anzai et al., 2012). They contribute to the recruitment and activation of immune cells and facilitate the release of inflammatory cytokines, thereby initiating immune responses. It is noteworthy that dendritic cell-derived small extracellular vesicles (DC-sEVs) are rich in a variety of miRNAs associated with angiogenesis, which endows them with great potential in promoting post-infarction vascular regeneration. Although research on DC-derived sEVs remains limited, existing findings further support their pro-angiogenic potential (Liu et al., 2016). In experimental studies, sEVs derived from mouse bone marrow-derived dendritic cells were administered in a murine MI model. Parallel *in vitro* experiments involved co-culture of these sEVs with rat cardiac microvascular ECs. The results showed that DC-sEVs effectively stimulated tube formation in cardiac microvascular ECs. In the MI model, these sEVs also upregulated the expression of VEGF and platelet ECs adhesion molecule CD31 within the infarcted myocardium, thereby robustly enhancing angiogenesis post-MI. Further mechanistic analysis revealed that miR-494-3p, carried by DC-sEVs infiltrating the infarcted heart, plays a key role in promoting angiogenesis and facilitating cardiac repair after MI (Liu et al., 2021). DC-sEVs can foster an ideal microenvironment for angiogenesis following myocardial infarction. In a mouse model of myocardial infarction, CCR7-modified DC-sEVs were shown to significantly enhance CD4^+^ T cell activation, promote M2 macrophage polarization, and increase the secretion of pro-angiogenic factors, such as vascular endothelial growth factor and angiopoietin-1, thereby promoting angiogenesis (Zhang et al., 2023). Separately, in an earlier study, Zhang et al. demonstrated that a hydrogel loaded with dendritic cell-derived extracellular vesicles could create a favorable microenvironment for angiogenesis by modulating regulatory T cells and macrophage polarization (Zhang Y. et al., 2021). Together, these findings underscore the potential of engineered DC-sEVs and advanced delivery systems to promote cardiac repair through immunomodulation. Given the currently limited research on DC-sEVs in promoting post-myocardial infarction angiogenesis, their potential as a therapeutic strategy for MI warrants further investigation.

#### 3.2.3 sEVs derived from blood

The bloodstream represents the most abundant reservoir of circulating sEVs, holding significant promise for the diagnosis and treatment of cardiovascular diseases. Based on post-collection processing, blood-derived sEVs are primarily categorized into plasma and serum subtypes. These two subgroups differ fundamentally in cellular origin, molecular composition, and functional properties due to their differential exposure to the *ex vivo* coagulation process. The precise discrimination and systematic comparison between plasma-sEVs and serum-sEVs are crucial for elucidating their biological roles and advancing the development of reliable biomarkers and standardized therapeutic preparations.

##### 3.2.3.1 sEVs derived from plasma

Plasma, the liquid component of blood obtained by centrifugation following collection with anticoagulants, contains a population of sEVs that originate from a broad spectrum of circulating blood cells—such as platelets, erythrocytes, and leukocytes—as well as vascular endothelial cells and other tissue-derived vesicles released into the circulation under physiological or pathological conditions. Since anticoagulation processing minimizes *ex vivo* coagulation activation, the composition of plasma-sEVs is considered to more faithfully mirror the *in vivo* pathophysiological state.

Studies have confirmed that plasma-sEVs carry abundant pro-angiogenic signals. For example, sEVs isolated from rats subjected to remote ischemic preconditioning can significantly upregulate the expression of key molecules such as eNOS, HIF-1α, and VEGF through a mechanism mediated by Hsp70, thereby enhancing endothelial function, promoting post-myocardial infarction angiogenesis, and improving cardiac function (Chen et al., 2020). Beyond direct effects, plasma-sEVs can also indirectly facilitate repair by modulating stem cell functions. Jin et al. co-cultured plasma-sEVs from post-MI rats with BM-MSCs before transplantation and found that these sEVs could reduce stem cell apoptosis by activating the AKT signaling pathway, promote endothelial differentiation, and significantly increase capillary density in the infarct zone, synergistically improving cardiac remodeling (Jin et al., 2021). CD44 has been identified as a key receptor regulating plasma-sEVs function. Zhang et al. demonstrated that the pro-angiogenic effect of plasma-sEVs after myocardial infarction depends on their internalization mediated by endothelial surface CD44, which subsequently enhances downstream FGFR2 signaling. Although this study did not specify the single cellular origin of the sEVs, it revealed that their functional efficacy relies on specific docking with receptors on target cells (Zhang et al., 2022b). Moreover, the functionality of plasma-sEVs is significantly influenced by donor physiological status. Wang et al. found that plasma sEVs from young healthy individuals are enriched with miR-664a-3p, which can target mothers against decapentaplegic homolog 4 (SMAD4) in cardiac fibroblasts, inhibit the transforming growth factor-beta (TGF-β) pathway, exert anti-fibrotic effects, and optimize the microenvironment for angiogenesis. This study highlights the functional “youthful” advantage of such sEVs and suggests that donor age should be considered a critical quality control and screening parameter in plasma sEV-based therapeutic strategies (Wang et al., 2025).

##### 3.2.3.2 sEVs derived from serum

Serum is the liquid component obtained after whole blood coagulation *in vitro*. Consequently, serum-sEVs are highly enriched in vesicles released by activated platelets during the coagulation process, while also containing sEVs originating from the clot, damaged endothelium, or other sources. This process inevitably modifies the composition of serum-sEVs through *ex vivo* coagulation events, yet simultaneously establishes them as a convenient window for studying platelet-associated pathologies and leveraging routinely available clinical samples.

Extensive research utilizing clinical serum samples has revealed the pro-angiogenic roles of serum-sEVs. For instance, miR-1956 delivered by serum-sEVs released from ischemic myocardial and renal tissues in MI patients promotes angiogenesis by modulating the Notch-1/VEGF paracrine signaling axis in adipose-derived mesenchymal stem cells (Gao et al., 2020b). Additionally, miR-126-3p in serum sEVs enhances ECs function by targeting tuberous sclerosis complex 1 (TSC1), activating the mTOR complex 1(mTORC1) pathway, and upregulating HIF-1α and VEGFA expression (Duan et al., 2022). Furthermore, the circular RNA circular rna cebpz opposite strand (circCEBPZOS) carried by serum sEVs promotes the proliferation and tube-forming capacity of vascular smooth muscle cells *via* the miR-1178-3p/PDPK1 axis (Yu L. et al., 2023). Notably, the molecular content and function of serum-sEVs are closely linked to disease status. Compared with healthy controls, levels of miR-143 are significantly reduced in coronary serum sEVs from MI patients; this miRNA promotes nitric oxide production and endothelial repair by activating the insulin-like growth factor 1 receptor (IGF-1R) pathway (Geng et al., 2020). More importantly, underlying comorbidities profoundly alter the functional properties of serum-sEVs. Studies demonstrate that angiopoietin-like protein 6 (ANGPTL6) in serum sEVs from patients with acute MI (AMI) alone effectively promotes angiogenesis *via* the MAPK pathway. In contrast, serum sEVs from patients with AMI complicated by diabetes (AMI-DM) lose this capacity and even exhibit anti-angiogenic properties, indicating that diabetes severely impairs the reparative function of sEVs (Wang et al., 2024). These findings establish the dual value of serum-sEVs as sensitive biomarkers of disease status and a source of potential therapeutic targets.

##### 3.2.3.3 Systematic comparison between plasma-sEVs and serum-sEVs

Although both plasma-sEVs and serum-sEVs possess pro-angiogenic potential, their inherent heterogeneity dictates distinct positioning in research and application. In terms of source and preparation, the fundamental difference lies in exposure to *ex vivo* coagulation: plasma-sEVs, obtained using anticoagulants, largely preserve the native profile of circulating vesicles *in vivo*, whereas serum-sEVs are products of the coagulation process and are predominantly derived from platelets. This fundamental distinction leads to divergences in molecular composition and functional characteristics. The molecular profile of plasma-sEVs is considered more complex and balanced, reflecting the status of a wider variety of cells such as endothelial and immune cells. In contrast, serum-sEVs are strongly enriched in platelet-specific markers (e.g., CD41, CD61) and molecules associated with coagulation and thrombosis. Consequently, their functions often exhibit a “double-edged sword” nature—combining pro-angiogenic effects with potential pro-inflammatory and pro-coagulant activities.

This compositional difference profoundly influences the biological significance of key regulatory factors. Taking ANGPTL6 and miR-143 as examples, the loss of ANGPTL6 function in serum sEVs from AMI-DM patients may result from combined effects of the diabetic pathological milieu and coagulation-associated modifications. Whether diabetes induces similar alterations in plasma-sEVs remains to be verified under conditions that exclude coagulation interference. Similarly, the reduction of miR-143 in serum sEVs from MI patients has an ambiguous cellular origin—it could stem from genuine decreased secretion by endothelial cells or from dilution by the abundant release of other sEVs from activated platelets. Therefore, biomarker studies based on serum sEVs require cautious interpretation regarding cellular origin.

Thus, plasma-sEVs and serum-sEVs each have distinct application profiles. Serum sEVs, due to easier sample availability and compatibility with routine clinical testing, are suitable for retrospective biomarker screening and preliminary mechanistic association studies. However, their platelet-dominated composition and functional heterogeneity limit their direct application as therapeutic products. In comparison, plasma-sEVs, which better retain the *in vivo* native state, are more appropriate for in-depth mechanistic investigation and the development of standardized therapeutic preparations. Future research should employ multi-omics comparisons, single-vesicle sorting, and related technologies to systematically elucidate the differences between the two and, based on the pathological requirements of different disease stages, promote their translation into precise clinical applications.

#### 3.2.4 sEVs derived from various non-stem cells: differences and similarities

**FIGURE 4:**
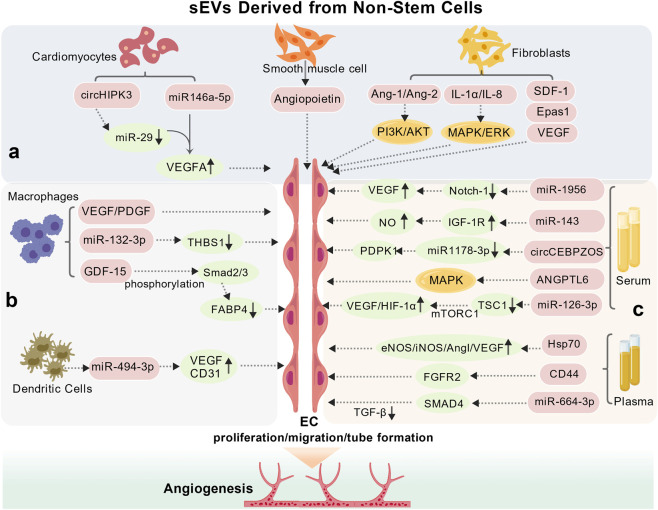
Mechanisms involved in the modulation of angiogenesis by Non-Stem Cell-derived sEVs. Cardiomyocyte-derived sEVs promote angiogenesis *via* circHIPK3/miR-146a-5p-mediated VEGFA upregulation. Smooth muscle cell-derived sEVs deliver angiopoietin to support ECs function. Fibroblast-derived sEVs enhance angiogenesis through SDF-1/Epas1/VEGF transfer and PI3K/Akt-MAPK/ERK activation *via* Ang-1/IL-1α/IL-8. Macrophage-derived sEVs act *via* VEGF/PDGF, miR-132-3p/THBS1 downregulation, and GDF-15/Smad2/3-mediated FABP4 inhibition. Dendritic cell-derived sEVs carry miR-494-3p to upregulate VEGF/CD31. Serum--sEVs deliver miR-1956/miR-143/miR-126-3p/circCEBPZOS/ANGPTL6, while plasma-d sEVs upregulate eNOS/iNOS/HIF-1α/Ang-1/VEGF *via* HSP70 and CD44/FGFR2 or SMAD4/TGF-β signaling to promote angiogenesis. circHIPK3, circular homeodomain-interacting protein kinase 3 RNA; Ang-1/Ang-2, angiopoietins; IL-1α, Interleukin 1-1α; IL-8, interleukin 8; SDF-1, stromal cell-derived factor-1; Epas1, endothelial pas domain protein 1; GDF-15, growth differentiation factor 15; THBS1, thrombospondin-1; Smad2/3, mothers against decapentaplegic homolog 2/3; FABP4, fatty acid-binding protein 4; IGF-1R, insulin-like growth factor 1 receptor; PDPK1, 3-Phosphoinositide-dependent protein kinase 1; circCEBPZOS, circular rna cebpz opposite strand; mTORC1, mTOR complex 1; TSC1, targeting tuberous sclerosis complex 1; ANGPTL6, angiopoietin-like protein 6; FGFR2, fibroblast growth factor receptor 2; CD44, Cluster of differentiation 44. SMAD4: mothers against decapentaplegic homolog 4; TGF-β: transforming growth factor-beta. **(a)** sEVs derived from cardiac cells; **(b)** sEVs derived from immune cells; **(c)** sEVs derived from blood.

Non-stem cell-derived sEVs from cardiac, immune, and blood sources significantly contribute to post-MI angiogenesis through context-dependent release of bioactive molecules. Key mediators include miRNAs (e.g., circHIPK3, miR-132-3p) and proteins (e.g., VEGF, SDF-1), which modulate pathways such as PI3K/Akt and MAPK/ERK to enhance EC function (Figure 4; Table 2).

**TABLE 2:** Summary of studies on non-stem cell-derived sEVs promoting angiogenesis after MI.

sEVs cell source	Key molecule(s)	Mechanism of action	Model validation	Relative advantages and disadvantages	References
Cardiomyocytes	CircHIPK 3	Inhibits miR-29a activity, increases VEGFA expression, promotes EC proliferation/migration/tube formation.	*In vitro*: HUVECs. *In vivo*: Mouse MI model.	Advantages: 1. High tissue specificity, signals highly relevant to cardiac repair; 2. Can respond to pathological stimuli like hypoxia, actively releasing reparative sEVs. Disadvantages: Extremely difficult to obtain from living subjects.	Wang et al. (2020)
	miR-146a-5p	Upregulates VEGFA expression, further increases ROS production, enhances infarct-related ER stress-mediated autophagy, promotes EC proliferation/migration/tube formation.	*In vitro*: Co-culture system of cardiomyocytes and macrophages or conditioned medium treatment.		Chen et al. (2022)
Cardiac fibroblasts	Ang-I, Ang-II, IL-1α, IL-8	Activates PI3K/AKT and MAPK/ERK signaling pathways, enhances EC proliferation/migration/tube formation.	*In vitro*: Co-culture system of human keratinocytes/fibroblasts with silk fibroin scaffold.	Advantages: 1. Abundant in the heart, key signaling source in the microenvironment; 2. Culture and engineering modifications (e.g., 3D culture) can optimize sEVs function. Disadvantages: Function presents a “double-edged sword” characteristic, coexistence of pro- and anti-angiogenic factors, complex regulation.	Hu et al. (2023)
	VEGE, SDF-1	Promotes angiogenesis, inhibits cardiomyocyte apoptosis.	*In vitro*: Cardiac fibroblasts. *In vivo*: Rat chronic MI model.		Tseliou E et al. (2015)
Vascular smooth muscle cells	Angiogenic factors	Effectively promotes EC proliferation/migration/tube formation.	*In vitro*: 3D silk fibroin scaffold culture system of human vascular smooth muscle cells.	Advantages: Directly related to vascular structure and homeostasis, signals have physiological relevance. Disadvantages: Studies mostly remain at the *in vitro* stage, incomplete evidence chain for *in vivo* efficacy.	Hu P. et al. (2022)
Macrophages	miR-132-3p	Downregulates THBS1 expression, enhances EC proliferation/migration/tube formation.	*In vitro*: HUVECs. *In vivo*: Mouse MI model.	Advantages: 1. Possesses dual powerful functions: Immunomodulation and pro-angiogenesis; 2. High phenotypic plasticity, sEVs function can be optimized *via* preconditioning (e.g., GDF-15 transfection). Disadvantages: Phenotype instability, M2 phenotype may revert to M1 *in vivo*.	Guo et al. (2024)
	GDF-15	Activates Smad2/3 phosphorylation, inhibits FABP4 production.	*In vitro*: Engineered sEVs surface-displaying macrophage-targeting peptide and GDF-15. *In vivo*: Rat acute MI model.		Xiao et al. (2024)
Dendritic cells	miR-494-3p	Upregulates VEGF and CD31 expression.	*In vitro*: Mouse BMDCs, rat CMECs. *In vivo*: Mouse MI model.	Advantages: As professional antigen-presenting cells, their sEVs may possess unique immunomodulatory properties. Disadvantages: Their immune-activating properties may carry uncontrollable inflammatory risks.	Liu et al. (2021)
Plasma	miRNA	Targets Hsp70, upregulates eNOS, iNOS, HIF-1α, Ang-I, VEGF expression.	*In vitro*: Rat CMECs. *In vivo*: Rat remote ischemic preconditioning model, rat MI model.	Advantages: 1. Non-invasive, easy repeated collection, shortest path for clinical translation; 2. Carries systemic, integrated protective signals (e.g., post-RIC). Disadvantages: 1. Extremely complex composition, mixture of sEVs from various cells, mechanism difficult to pinpoint; 2. Significant donor individual variation (age, disease) affects sEVs efficacy.	Chen et al. (2020)
	miRNA	Activates CD44/FGFR2 pathway, promotes EC proliferation/migration/tube formation.	*In vitro*: Co-culture of HUVECs/mouse primary MVECs with mouse plasma exosomes simulating ischemic microenvironment. *In vivo*: Mouse MI model.		Zhang et al. (2022b)
	miR-664a-3p	Targets SMAD4, inhibits TGF-β pathway.	*In vitro*: Mouse primary cardiac fibroblasts, TGF-β1-induced fibrosis. *In vivo*: Mouse MI model.		Wang et al. (2025)
Serum	miR-1956	Downregulates Notch1, activates VEGF signaling.	*In vitro*: Peripheral serum from acute myocardial infarction (AMI) patients and healthy volunteers, HUVECs. *In vivo*: Mouse MI model.	Advantages: Rich in platelet activation signals, may be suitable for specific repair stages. Disadvantages 1. Platelet-derived sEVs dominate, with functions skewed toward coagulation and inflammation, resulting in high “noise” 2. The *in vitro* coagulation process severely interferes with the original composition of sEVs, necessitating caution when extrapolating conclusions to *in vivo* settings.	Gao et al. (2020a)
	miR-143	Activates IGF-1R, increases NO production.	*In vitro*: Coronary artery serum from AMI patients. *In vivo*: Mouse MI model.		Geng et al. (2020)
	ANGPTL6	Activates MAPK pathway.	*In vitro*: HUVECs, mouse/rat primary CMECs. *In vivo*: Diabetic mouse and MI mouse models.		Wang et al. (2024)
	miR-126-3p	Targets TSC1 expression, activates mTORC1 signaling, increases HIF-1α and VEGF expression.	*In vitro*: Co-culture of serum exosomes from AMI patients (AMI-exo) and healthy controls (con-exo) with HUVECs. *In vivo*: Mouse hindlimb ischemia and acute MI models.		Duan et al. (2022)
	circCEBPZOS	Activates miR-1178-3p/PDPK1 pathway, promotes smooth muscle cell proliferation/migration/tube formation.	*In vitro*: Hypoxia/reoxygenation of cardiac microvascular endothelial cells. *In vivo*: Mouse MI model.		Yu L. et al. (2023)

Different sEVs sources each possess distinct advantages: cardiomyocyte-derived sEVs regulate oxidative stress and inflammation; fibroblast-derived sEVs exhibit dual roles influenced by LYPD-1 and Epas1; SMC- derived sEVs support vascular stability; macrophage-derived sEVs promote angiogenesis *via* M2 polarization; DC-sEVs require further investigation; Plasma-sEVs and serum-derived sEVs reflect pathological states and offer diagnostic and therapeutic potential.

Several limitations remain. First, the regulatory mechanisms governing the pro- and anti-angiogenic balance within cardiac fibroblast-derived sEVs remain poorly understood, and specific strategies to inhibit LYPD-1 are lacking. Second, immune cell-derived sEVs exhibit phenotype instability (e.g., M2 macrophage polarization), challenging large-scale production. Third, blood-derived sEVs show considerable inter-individual variability, and methods to restore functionality under disease conditions are absent.

To address these challenges, future studies should focus on: (1) Genetic editing of LYPD-1 in fibroblast-derived sEVs to modulate angiogenic balance; (2) Optimization of induction protocols for maintaining stable M2 macrophage phenotypes; (3) Enhancing pro-angiogenic activity in serum-derived sEVs from comorbid patients (e.g., *via* ANGPTL6 supplementation); (4) Exploring combination strategies (e.g., cardiac cell- and blood-derived sEVs); to improve targeting and bioavailability.

## 4 Engineered sEVs delivery strategies for MI therapy

sEVs serve as key mediators of intercellular communication and play an important role in post-myocardial infarction tissue repair by delivering bioactive molecules, demonstrating considerable therapeutic potential. However, natural sEVs face limitations such as weak targeting specificity, short *in vivo* retention time, and rapid clearance, which hinder their clinical translation (Ma et al., 2024; Kim et al., 2024). In recent years, engineering strategies to modify sEVs have emerged as a vital approach to enhance their targeting ability, prolong circulation time, and improve therapeutic efficacy (Yoo et al., 2025; Erana-Perez et al., 2024). This section systematically reviews major engineering strategies, including hydrogel encapsulation, peptide-modified biomimetic carriers, membrane fusion technology, and microneedle patches, and compares them in terms of targeting capability, myocardial retention, circulation time, and translational feasibility, thereby elucidating the impact of different delivery strategies on the therapeutic outcomes of myocardial infarction.

### 4.1 Hydrogel encapsulation strategy

As hydrophilic polymers with a three-dimensional network structure, hydrogels can encapsulate sEVs and achieve their controlled and sustained release. Commonly used materials include natural polysaccharides (e.g., alginate, hyaluronic acid) and proteins (e.g., collagen, gelatin) (Ju et al., 2022). Studies have shown that loading sEVs into alginate gel to form an sEVs-Gel composite system significantly prolongs the retention time of sEVs in myocardial tissue, enhances their promotive effects on angiogenesis and macrophage polarization, and improves cardiac function while reducing fibrosis (Lv et al., 2019). Han et al. developed an injectable self-assembling hydrogel that incorporates a cardioprotective peptide and a matrix metalloproteinase-2 (MMP2)-responsive sequence for sEVs encapsulation. This system demonstrated superior anti-inflammatory, anti-apoptotic, and functional recovery effects compared to sEVs alone after myocardial infarction (Han Y. et al., 2019). Further studies have demonstrated that an injectable hyaluronic acid-polylysine hydrogel loaded with sEVs can more effectively promote myocardial repair by synergistically modulating the oxidative stress and inflammatory microenvironment (Ren et al., 2024). Furthermore, Chen et al. prepared a shear-thinning hydrogel capable of sustained sEVs release for over 21 days, further confirming the role of hydrogels in maintaining long-term therapeutic efficacy (Chen et al., 2018).

### 4.2 Microneedle patches

As a minimally invasive transdermal delivery system, microneedle patches enable the local and sustained release of sEVs into myocardial tissue. Yuan et al. loaded sEVs carrying miR-29b onto a gelatin-based microneedle patch and implanted it onto the cardiac surface of mice following myocardial infarction. This approach significantly prolonged the retention of sEVs in the infarcted area, suppressed fibrosis, and alleviated inflammation (Yuan et al., 2023). Similarly, a porous microneedle patch developed by Fang et al. achieved sustained sEVs release for at least 7 days, promoting angiogenesis and functional recovery in a spinal cord injury model (Fang et al., 2023). This strategy offers a feasible approach for non-invasive and long-term cardiac repair.

### 4.3 Membrane fusion technology

This technique involves fusing sEVs with functional liposomes or cell membranes, endowing the resulting hybrid vesicles with specific targeting molecules and enhanced stability. Li et al. fused platelet membranes with sEVs to construct platelet membrane-modified EVs (P-EVs), which leverage platelet-monocyte interactions to be selectively recruited to myocardial injury sites, effectively promoting repair in an ischemia-reperfusion model (Li et al., 2022). The membrane fusion strategy not only improves the targeting capability of sEVs but also extends their circulation time, offering a novel approach for precise delivery.

### 4.4 Peptide-modified biomimetic carrier strategy

The surface modification of sEVs with specific targeting peptides can significantly enhance their homing ability to ischemic myocardium. Utilizing a hydrophobic insertion method, Chen et al. displayed a cardiac-targeting peptide (CTP) on the sEVs membrane, constructing CTP-EVs that efficiently delivered curcumin to the infarcted area and promoted the recovery of cardiac function (Chen et al., 2023). In another approach, Zhang et al. designed a biomimetic nanocapsule system using umbilical cord mesenchymal stem cell-derived sEVs as carriers. This system was internally loaded with placental growth factor (PLGF) and surface-modified *via* covalent conjugation of a cardiac-homing peptide (CHP). This engineered platform demonstrated significant effects in promoting angiogenesis, reducing fibrosis, and improving cardiac function in animal models (Zhang et al., 2024a).

### 4.5 Comparison of the four strategies

In summary, different engineering strategies provide complementary solutions for the clinical application of sEVs (Table 3). The core advantages of hydrogel and microneedle patch strategies lie in achieving long-term local retention and controlled release of sEVs at the lesion site, with clear prospects for translational feasibility. In contrast, peptide modification and membrane fusion strategies focus on enhancing systemic active targeting capability and circulatory stability to achieve efficient “targeted accumulation,” although their production processes face greater challenges. Future development trends favor the construction of composite engineering systems (e.g., loading targeting peptide-modified sEVs into smart responsive hydrogels or microneedles), thereby synergistically optimizing targeting, retention, release, and pharmacokinetics to maximize therapeutic efficacy and accelerate clinical translation.

**TABLE 3:** Systematic comparison of four sEVs engineering strategies.

Strategy	Targeting capability	Retention	Circulation time	Translational feasibility
Hydrogel encapsulation strategy	Passive targeting, relying on local injection for site-specific delivery.	Exceptional, achieved through physical entrapment to enable long-term sustained release and retention.	Significantly enhances local sustained release, with no marked improvement in systemic circulation time.	High, owing to good material biocompatibility and injectable formulations that facilitate clinical translation.
Microneedle patches	Local/transdermal targeting, achieved *via* minimally invasive implantation for direct delivery to the lesion site.	Strong, as the patch provides physical anchoring to enable sustained local release.	Primarily characterized by long-term localized release, exerting minimal impact on systemic circulation duration.	High, due to its minimally invasive nature, good patient compliance, and relatively mature manufacturing processes.
Membrane fusion technology	Active/biomimetic targeting, leveraging the inherent targeting properties of fused membranes (e.g., platelet membranes).	Relatively strong, as the biomimetic properties enhance adhesion and retention at injury sites.	Significantly prolonged, as the hybrid membrane structure enhances *in vivo* stability.	Moderate to low, as membrane extraction and fusion processes are complex, posing significant quality control challenges.
Peptide-modified biomimetic carrier strategy	Active targeting, achieved through specific peptides (e.g., CTP), enables precise homing.	Moderate, dependent on targeting ligand-receptor interactions to enhance accumulation at the lesion site.	Markedly extended, as surface modifications aid in evading immune clearance.	Moderate, where standardization of targeting peptide synthesis and conjugation processes is critical.

## 5 Challenges and future research directions

### 5.1 Clinical translation challenges

The therapeutic potential of sEVs for MI has been extensively validated. sEVs can carry various bioactive molecules and promote angiogenesis and cardiac repair by modulating key signaling pathways. For instance, sEVs enriched with miR-126, miR-1956, and others can activate the PI3K/Akt and VEGF pathways, thereby enhancing endothelial cell proliferation and migration (Gao et al., 2020b; Zhang L. et al., 2021). Concurrently, proteins carried by sEVs, such as Ang-I and HIF-1α, can improve vascular stability and permeability, supporting myocardial repair and cardiac function improvement (Gong et al., 2017; Ibrahim and Marbán, 2016). Consequently, sEVs can serve not only as directly administrable therapeutic agents but also be engineered as targeted delivery vehicles, offering novel avenues for restoring endothelial function. In recent years, sEVs derived from Chinese herbal medicines (CHM-sEVs) have emerged as a research hotspot in the integrated approach of traditional Chinese and Western medicine for MI treatment, owing to their natural active components and endogenous regulatory capabilities (Zhang et al., 2024b; Fu et al., 2025; Peng et al., 2025). For example, sEVs derived from Salvia miltiorrhiza contain characteristic components like tanshinone and salvianolic acid, which significantly enhance endothelial cell function and effectively promote new blood vessel formation in both the chick embryo chorioallantoic membrane assay and mouse ischemic models (Zhang S. et al., 2024). sEVs from *Rhodiola rosea* can promote angiogenesis in ischemic tissues by inhibiting the TXNIP/NLRP3 signaling pathway, thereby reducing vascular endothelial cell pyroptosis (Liu et al., 2025). These studies highlight the unique advantages of CHM-sEVs in regulating angiogenesis and myocardial repair. Therefore, sEVs, particularly those of herbal origin, represent a versatile biological agent capable of directly modulating angiogenesis and can also be engineered into targeted carriers for delivering therapeutic drugs. This dual functionality provides new strategic possibilities for the precise treatment of myocardial infarction.

**FIGURE 5:**
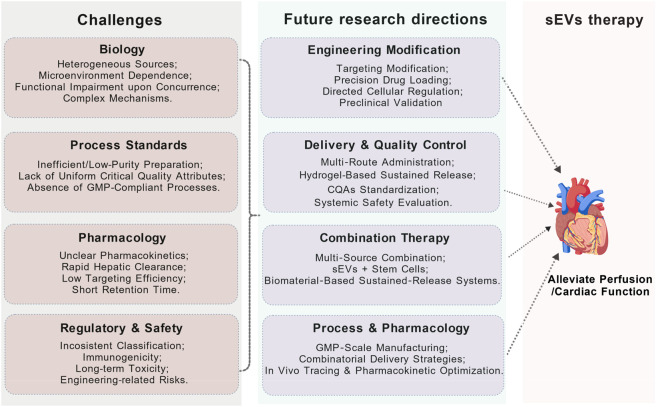
Challenges and future research directions of sEVs in treating MI. The clinical translation of sEVs-based pro-angiogenic therapy faces four major challenges: biological and functional heterogeneity, manufacturing bottlenecks, inefficient pharmacokinetics and delivery, as well as regulatory and safety concerns. In response, four future research directions are proposed, including optimization of engineering strategies, improvement of delivery systems and quality control, development of combination therapies, and breakthroughs in scalable production and pharmacological understanding. These efforts aim to advance this therapeutic approach from basic research to clinical application.

Despite the promising prospects, pro-angiogenic therapies based on sEVs face a series of formidable challenges on the path to clinical translation (Figure 5). First, challenges arise from the heterogeneity and complexity of their biological functions. The functionality of sEVs is highly dependent on their cellular origin and the state of the microenvironment. For instance, sEVs derived from M1-polarized macrophages can impair angiogenesis by delivering miR-155, which suppresses endothelial cell function (Liu et al., 2020). Similarly, cardiomyocyte-derived sEVs carrying miR-19a-3p inhibit endothelial cell function by targeting HIF-1α), worsening outcomes in post-myocardial infarction mice (Gou et al., 2020). More critically, under comorbid conditions such as diabetes, the vascular repair function of patients’ endogenous sEVs is compromised. This presents a fundamental challenge for autologous sEVs-based therapies, potentially necessitating a shift towards allogeneic or engineered sources (Wang et al., 2024).

Second, technical and manufacturing bottlenecks hinder their pharmaceutical development. Current mainstream laboratory preparation methods, such as ultracentrifugation, suffer from low efficiency and poor purity, making them inadequate for pharmaceutical-grade production (Ailuno et al., 2020). Furthermore, the lack of unified and actionable standards for critical quality attributes (CQAs) across the industry remains a core obstacle impeding the transition from research to clinical application.

Third, pharmacokinetics and delivery efficiency pose dual pharmacological challenges. The *in vivo* behavior of sEVs remains poorly defined, with unclear biodistribution and half-life. Systemic administration faces significant hurdles, including rapid hepatic clearance, extremely low cardiac targeting efficiency, and short retention time at the lesion site, which severely obstructs the development of rational dosing regimens (Herrmann et al., 2021).

Finally, ambiguous regulatory pathways and long-term safety risks constitute the ultimate barriers to clinical translation. The global regulatory framework is not yet unified, with regional disparities in their classification (as biological products or advanced therapy medicinal products) creating uncertainty for approval. Regarding safety, a systematic evaluation is required for the immunogenicity of allogeneic sEVs, potential long-term toxicity due to non-target organ accumulation, and off-target or carcinogenic risks associated with engineered loading of exogenous active substances (Zhu et al., 2017). Clarifying the regulatory path and establishing a comprehensive long-term safety evaluation system are prerequisites for advancing sEVs-based therapies into clinical application.

### 5.2 Future research directions

To overcome the aforementioned translational challenges, future research must focus on several key multidimensional areas to systematically advance sEVs-based therapies toward clinical application (Figure 5). First, optimizing engineering strategies and strengthening preclinical validation is essential. Beyond conventional peptide modifications for enhanced targeting (Vandergriff et al., 2018; Wang et al., 2018), future efforts should prioritize the precise delivery of specific therapeutic molecules (e.g., miRNAs, proteins) through parental cell genetic engineering or direct post-isolation loading. Preclinical studies have confirmed the feasibility of this strategy. For example, engineered sEVs displaying a cardiac-targeting peptide on their surface and loaded internally with curcumin have been shown to achieve efficient targeting of infarcted myocardium, thereby promoting cardiac functional recovery (Chen et al., 2023). Furthermore, Yu et al. utilized silicate ions to activate endothelial progenitor cells, inducing the secretion of sEVs enriched with miR-126-3p. These sEVs improved post-infarction cardiac functional recovery by promoting angiogenesis and inhibiting apoptosis (Yu B. et al., 2023).

Second, dose optimization and safety evaluation constitute the core of clinical translation for sEVs-based therapies. Dosing regimens must be personalized, integrating factors such as the sEVs source, engineering strategies, and the administration route. Local sustained-release delivery systems, for instance *via* hydrogels, help minimize systemic dosage and off-target risks. For systemic administration, intravenous injection necessitates engineering approaches, such as surface modifications, to address the conflict between rapid hepatic clearance and therapeutic efficacy. Concurrently, emerging delivery routes—such as inhalable administration, exemplified by stem cell-derived exosomes that promote post-myocardial infarction cardiac repair through efficient pulmonary absorption and systemic distribution—offer a promising non-invasive alternative, potentially mitigating rapid clearance challenges (Li J. et al., 2024b). Establishing Critical Quality Attributes (CQAs) based on biological potency, such as specific miRNA expression profiles, is essential for achieving dose standardization and batch-to-batch consistency. Safety assessments must systematically address the immunogenicity of allogeneic sEVs, potential toxicity from non-target organ accumulation, and off-target risks of engineered cargo. The evaluation framework can draw on experiences from completed clinical trials for other conditions. For example, early-phase trials of sEVs in Acute Respiratory Distress Syndrome (Shi et al., 2021) and Congenital Myasthenic Syndromes (Prodromos et al., 2025) have preliminarily demonstrated clinical safety and feasibility, providing valuable safety data for cardiovascular translation.

Third, exploring synergistic effects between sEVs and other therapeutic modalities is crucial. This can be achieved *via* three primary pathways: co-administering sEVs from complementary cell sources; combining sEVs with stem cells to enhance engraftment efficacy; or embedding sEVs into biomaterial hydrogels to create a local sustained-release system that addresses both the need for prolonged delivery and mechanical support for the infarcted heart (Hu X. et al., 2022). Preclinical studies have validated this strategy’s value. For example, Lv et al. constructed an sEVs-Gel composite system by loading sEVs into an alginate hydrogel, which significantly prolonged sEV retention in the myocardial area, enhanced their promotion of angiogenesis and macrophage polarization, and effectively improved cardiac function while reducing fibrosis (Lv et al., 2019).

Fourth, breakthroughs in technical production and pharmacological bottlenecks are essential. On the production front, it is necessary to establish GMP-compliant, scalable processes based on technologies like tangential flow filtration and to define CQAs encompassing particle characteristics, protein markers, biological potency, and nucleic acid content to ensure batch-to-batch consistency. Pharmacologically, to address issues of low delivery efficiency and short half-life *in vivo*, a synergistic strategy combining “engineering modifications + hydrogel loading + minimally invasive local administration” can be employed. Peptide modifications confer cardiac targeting, hydrogels create a local sustained-release reservoir, and minimally invasive delivery routes (e.g., intramyocardial or intrapericardial injection) minimize systemic clearance, enabling precise and efficient therapy.

In summary, the clinical translation of sEVs-based therapies requires a stepwise strategy, beginning with dose, efficacy, and safety evaluation in large animal models of MI before advancing to confirmatory clinical trials. While the mechanisms by which sEVs promote post-infarct angiogenesis have been preliminarily elucidated, their core therapeutic efficacy stems from the therapeutic reprogramming of protein synthesis networks in target cells—a biological principle analogous to pathogen-mediated hijacking of host translational machinery. Future research should extend beyond the description of isolated signaling pathways. It must integrate multidisciplinary technologies to optimize sEVs characterization, targeting precision, and local retention, thereby mimicking and advancing programmable precise delivery. Evolving sEVs from mere carriers of bioactive molecules into programmable cell-instructive systems holds promise for overcoming translational barriers and establishing them as transformative therapies for MI and other cardiovascular diseases.
